# Increased Expression of Visfatin in Monocytes and Macrophages in Male Acute Myocardial Infarction Patients

**DOI:** 10.1155/2012/469852

**Published:** 2012-12-13

**Authors:** Cheng-An Chiu, Teng-Hung Yu, Wei-Chin Hung, Li-Fen Lu, Fu-Mei Chung, I-Ting Tsai, Chih-Ying Yang, Chia-Chang Hsu, Yung-Chuan Lu, Jer-Yiing Houng, Yau-Jiunn Lee, Chao-Ping Wang

**Affiliations:** ^1^Division of Cardiology, Department of Internal Medicine, E-Da Hospital, I-Shou University, No. 1 Yi-Da Road, Jiau-Shu Village, Yan-Chao District, Kaohsiung 82445, Taiwan; ^2^Division of Cardiac Surgery, Department of Surgery, E-Da Hospital, I-Shou University, No. 1 Yi-Da Road, Jiau-Shu Village, Yan-Chao District, Kaohsiung 82445, Taiwan; ^3^Department of Emergency, E-Da Hospital, I-Shou University, No. 1 Yi-Da Road, Jiau-Shu Village, Yan-Chao District, Kaohsiung 82445, Taiwan; ^4^Division of Gastroenterology and Hepatology, Department of Internal Medicine, E-Da Hospital, I-Shou University, No. 1 Yi-Da Road, Jiau-Shu Village, Yan-Chao District, Kaohsiung 82445, Taiwan; ^5^Division of Endocrinology and Metabolism, Department of Internal Medicine, E-Da Hospital, I-Shou University, No. 1 Yi-Da Road, Jiau-Shu Village, Yan-Chao District, Kaohsiung 82445, Taiwan; ^6^Department of Medical Nutrition, Institute of Biotechnology and Chemical Engineering, I-Shou University, No. 6 Yi-Da Road, Jiau-Shu Village, Yan-Chao District, Kaohsiung 82445, Taiwan; ^7^Lee's Endocrinologic Clinic, No. 130 Min-Tzu Road, Pingtung, 90000, Taiwan

## Abstract

We demonstrated that visfatin expressed in monocytes and neutrophils and increased their reactivity in male acute ST-segment elevation myocardial infarction patients. Furthermore, visfatin was strongly appeared in lipid rich coronary rupture plaques and macrophages. These results suggest another explanation about leukocytes mediated visfatin that may play a pathogenesis role in coronary vulnerable plaques rupture.

## 1. Introduction

As we know, acute myocardial infarction (AMI) constitutes the major cause of death in most countries and remains substantial in the years thereafter. Inflammation is implicated in the development and rupture of atheromatous plaques, and there is considerable evidence supporting the involvement of adipocytokines in this inflammatory process [1–3]. The recruitment of circulating monocytes into the arterial wall, followed by their differentiations into tissue macrophages, is one of the earliest events in atherosclerotic plaque formation [4–8]. Increasing evidence suggests that monocytes control vascular smooth muscle cells proliferation and migration [9–12], lipid metabolism [13], and inflammation [14–17] within the vessel wall. Monocytes have thus been proposed to serve as markers, initiators and promoters of arterial occlusive diseases [18, 19]. Hence, the phenomenon of leukocytosis might contribute to the development of chronic heart disease via multiple pathophysiological mechanisms, including vascular inflammation, proteolytic, and oxidative damage to the endothelial cells, plugging the microvasculature hypercoagulability, and promoting infarct expansion [20]. 

Visfatin, also known as pre-B-cell colony-enhancing factor, is a 52- to 55-kDa protein that is suggested to be highly prevalent in visceral fat cells. Researchers demonstrated that visfatin is a harmful, proinflammatory agent in obesity-induced metabolic and cardiovascular diseases [21, 22]. Clinical and basic reports have suggested that visfatin is an inflammatory protein associated with plaque destabilization and acute coronary syndrome (ACS) [23, 24]. Our recent studies also showed that plasma visfatin levels are associated with infarct-related artery (IRA) occlusion and that increased plasma visfatin may be closely related to the degree of myocardial damage [25, 26]. In addition, vulnerable lesions with a tendency to rupture are rich in activated macrophages, suggesting the macrophage as a key regulator of atherosclerotic plaque stability [27, 28]. Interestingly, visfatin has also been shown to be produced by immune cells (e.g., neutrophils and macrophages) [29, 30]. However, the expression of visfatin in monocytes and macrophage in circulation and coronary rupture plaques in patients with acute ST-segment elevation myocardial infarction (STEMI) has not been well elucidated.

In view of the possible association of visfatin with inflammation and pathogenesis of AMI, we thus studied the expression of visfatin in circulating leukocytes in male patients with STEMI by immunohistochemical (IHC) staining, flow-cytometry analysis, and real-time PCR. Furthermore, we also studied immunoreactivity of visfatin expression in macrophages in human coronary rupture plaques.

## 2. Materials and Methods

### 2.1. Study Population

We initially enrolled 7 consecutive male patients (age, 63 ± 14 years) admitted with a diagnosis of STEMI within 12 h of symptom onset, between June 2011 and June 2012. STEMI was indicated by prolonged chest pain (>30 min), typical rise increase in the levels of biochemical markers (Troponin-I and CK-MB/CPK) with ischemic symptoms lasting for ≥30 minutes, and an ST-segment elevation of ≥2.0 mm in ≥2 contiguous electrocardiographic leads. Six male non-CAD control subjects (age, 58 ± 13 years) had undergone a coronary angiography examination, and documented insignificant coronary stenosis was documented. Written informed consents were obtained from the patients before enrollment. The study was conducted in agreement with the guidelines approved by the Human Research Ethics Committee at our hospital.

### 2.2. Immunohistochemistry in Circulating Leukocytes

An avidin-biotin-peroxidase complex commercial method (DAKO Co., Carpinteria, CA) was used for IHC analysis. Approximately 100 *μ*L heparin-containing bloods were incubated with 2 mL of red blood cell (RBC) lysing solution (BD Biosciences, San Jose, CA) at room temperature for 15 min to remove RBCs. After washing with 2 mL of DPBS (Gibco, Grand Island, NY) containing 5% fetal bovine serum, the cells were resuspended in 0.1 mL of the same buffer and were centrifuged at 250 rpm for 8 min to the poly-L-lysine (Sigma chemical Co., St. Louis, MO), precoated slide by cytospin (Kubota-5920, Kubota Co., Tokyo). The cells on the slide were fixed with 95% methanal for 3 min at room temperature. Endogenous peroxidase activity was blocked by incubation of the sections in 0.3% H_2_O_2_ in phosphate-buffered saline (PBS) for 30 min and incubated with 1% bovine serum albumin (BSA) for 30 min to block nonspecific staining. Slides were drained and incubated overnight at room temperature in a humidity chamber with the respective rabbit anti-human primary antibody for visfatin (1 : 200) diluted with antibody diluent (DAKO Corp., Glostrup, Denmark). The primary antibody was purchased from Phoenix Pharmaceuticals Inc. (Belmont, CA). The avidin-biotin-peroxidase complex (ABC complex; DAKO, Carpinteria, CA, USA) was applied on the sections after they were incubated with biotinylated secondary antibody (Dako Corporation, Carpinteria, CA, USA). The slides were incubated with DAB substrate-chromogen solution (Dako Corporation, Carpinteria, CA, USA), counterstained with hematoxylin, and mounted in an aqueous mounting medium. Negative control studies were performed with replacement of the primary antibody by nonimmune antiserum and counterstaining with hematoxylin. 

### 2.3. Intracellular Staining and Flow-Cytometry

For double immunofluorescence staining, 100 *μ*L of heparin-containing blood was incubated with PE/CY5-conjugated mouse anti-human CD3 for T lymphocyte, PE-conjugated mouse anti-human CD13 for neutrophil, PE-conjugated mouse anti-human CD14 for monocyte, and PE/CY5 conjugated mouse anti-human CD19 for B lymphocyte, in the dark room for 30 min at room temperature. All the antibodies using for double immunofluorescence staining were purchased from BD Biosciences. Then, 2 mL of RBC lysing solution was added and incubated for 15 min at room temperature. After washing with 2 mL of DPBS containing 1% fetal bovine serum and 2% sodium azide, the cells were fixed with 3% paraformaldehyde in DPBS for 20 min at room temperature. The cells were washed two times with DPBS containing 0.05% saponin (Sigma), and then incubated with the primary rabbit anti-human PBEF/visfatin serum (Phoenix), which is diluted 100-folds with DPBS-saponin, for 20 min at room temperature. After washed by DPBS-saponin, cells were incubated with Alexa fluor 488 goat anti-rabbit IgG (Invitrogen/Molecular Probes) for 20 min at room temperature. Cells were gated based on forward angle light scatter and side angle light scatter and further analyzed using the Cell Quest Pro software (Becton Dickinson) for the expression of visfatin on peripheral blood cells.

### 2.4. Visfatin and CD68 Immunohistochemistry Stain of Coronary Rupture Plaques

The coronary rupture plaques were available from 7 of STEMI study patients who underwent primary percutaneous coronary intervention (PCI) and were aspirated by Medtronic Guard Wire and Aspiration System device as previously described [24]. An avidin-biotin-peroxidase complex commercial method (ABC complex; Dako Corporation, Carpinteria, CA, USA) was used for IHC analysis. In human coronary rupture plaques were 4-*μ*m-thick paraffin sections mounted on slides, dried for 30 min in an oven (60–70°C), and deparaffinized in xylene. The slides were pretreated with microwave heating as described previously [31]. After microwave treatment, the sections were washed in PBS. After this, endogenous peroxidase activity was blocked by incubation of the sections in 0.3% H_2_O_2_ in PBS for 20 min and incubated with 1% BSA for 30 min to block nonspecific staining. Sections were drained and incubated overnight at room temperature in a humidity chamber with the respective rabbit anti-human primary antibody for visfatin (1 : 200) and mouse anti-human primary antibody for CD68 (1 : 50) diluted with antibody diluent (DAKO Corp., Glostrup, Denmark). The ABC complex was applied on the sections after they were incubated with biotinylated secondary antibody. The sections were incubated with DAB substrate-chromogen solution (Dako Corporation, Carpinteria, CA, USA), counterstained with hematoxylin, and mounted in an aqueous mounting medium. Negative control studies were performed with replacement of the primary antibody by nonimmune antiserum and counterstaining with hematoxylin.

### 2.5. Circulating Leukocytes Visfatin mRNA Expression

Total circulating leukocytes RNA were isolated from the blood of 7 randomized AMI study patients and 6 non-CAD controls using TRIzol reagent. One microgram of each total-tissue RNA was diluted in water in a final volume of 50 *μ*L, and the RNA was reverse transcribed using the high-capacity cDNA archive kit (Applied Biosystems) in a 50 *μ*L reaction mixture. The RT reaction was carried out in the GeneAmp PCR System 9700 thermal cycler in two incubation steps, an initial 25°C incubation for 10 min followed by a final 37°C incubation for 2 h. Real-time PCR analysis was performed using a lightCycler1.5 Instrument (Roche, Mannheim, Germany). PCR was performed in a LightCycler capillary in a 10 *μ*L reaction volume that contained 1x DNA Master SYBR Green I, 2.5 mM MgCl_2_, 1 *μ*L cDNA, and 0.4 *μ*M primers. The PCR protocol was as follows: initial denaturation for 2 minutes at 95°C, 45 cycles at 95°C for 10 seconds, 60°C for 5 seconds, and 72°C for 12 seconds. Results were analyzed with LightCycler Software, version 3.5.3. Sequence-specific primers for Visfatin were a forward primer, CATAGGAGCATCTGCTCACTT and a reverse primer, GCTGCTGGAACAGAATAGCC. Sequence-specific primers for *β*-actin were a forward primer, TCCTTCCTGGGCATGGAGTC and a reverse primer, 5′-TTCTGCATCCTGTCGGCAATG-3′.

### 2.6. Statistical Analysis

The differences of visfatin mRNA expression between acute STEMI and control subjects were analyzed using Nonparametric Wilcoxon test. A *P* value less than 0.05 was considered statistically significant. The statistical analyses were performed using SAS statistical software, version 8.2 (SAS Institute Inc.; Cary, NC).

## 3. Results

**Figure 1:**
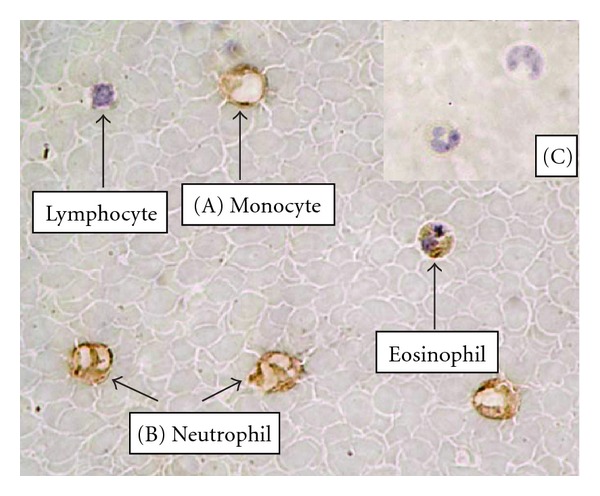
Immunohistochemical analysis of visfatin protein in WBC cells showed positive brown staining evident of monocyte (A) and neutrophil (B) (magnification, ×1000). Staining was absent in the control section, in which the primary antibody was replaced with nonimmune antiserum (C; magnification, ×400).

**Figure 2:**
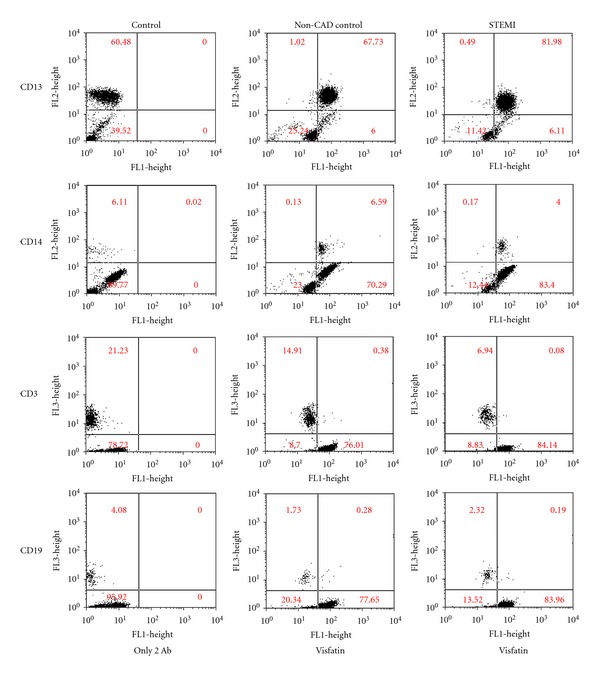
The intracellular staining for differentiating the different WBC counts by gating based on forward angle light scatter and side angle light scatter analysis. The strong staining on most neutrophil (CD13+) and monocyte (CD14+) from all subjects, but near no staining on T (CD3+) or B (CD19+) lymphocyte.

IHC analysis of visfatin protein in white blood cells (WBCs) showed positive brown staining evident of monocyte (Figure 1(A)) and neutrophil (Figure 1(B)) (magnification, ×1000). Staining was absent in the control section, in which the primary antibody was replaced with nonimmune antiserum (Figure 1(C); magnification, ×400).  Figure 2 showed that the intracellular staining for differentiating the different WBC counts by gating based on forward angle light scatter and side angle light scatter analysis. The strong staining on most neutrophil (CD13+) and monocyte (CD14+) from all subjects, but near no staining on T (CD3+) or B (CD19+) lymphocyte. 

**Figure 3:**
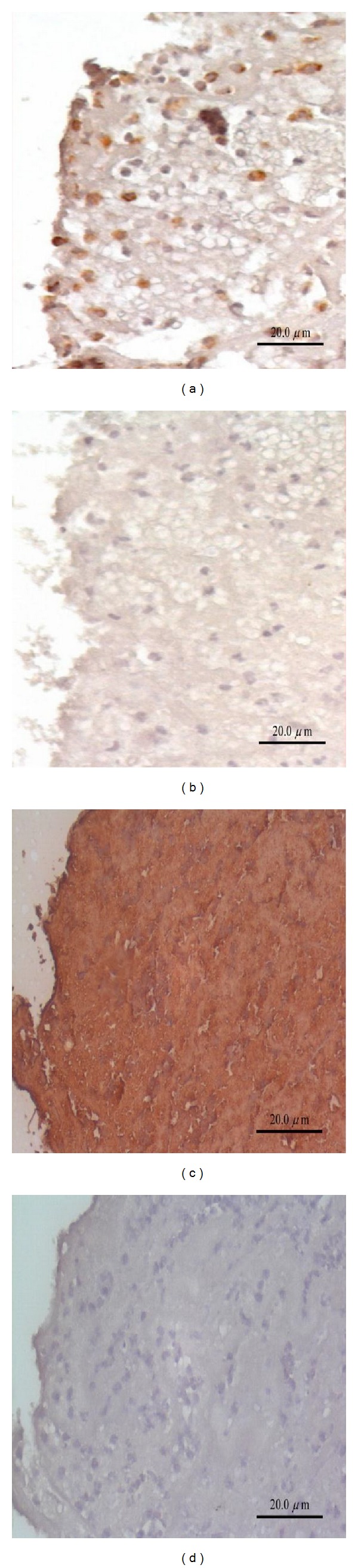
CD68 immunoreactivity of macrophages in coronary rupture plaques were noted (a; magnification, 20x), and strong visfatin immunoreactivity in atherosclerotic coronary rupture plaques in patients with STEMI was found (c; magnification, 20x). Absence of staining was found in the control section when the primary antibody was replaced with nonimmune antiserum (b and d).

**Figure 4:**
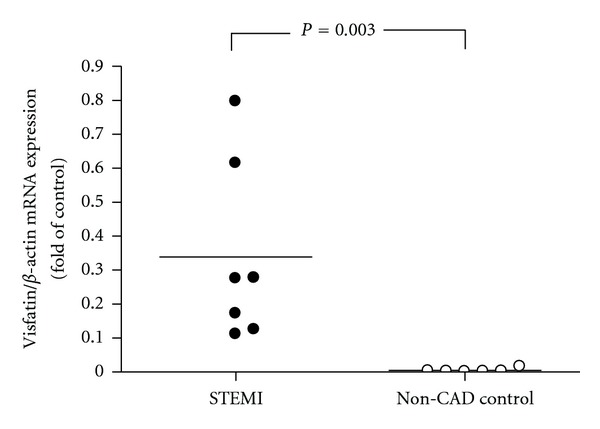
Individual values of visfatin mRNA expression according to disease status, the subjects were classified as having STEMI or being non-CAD controls. Data are expressed as the individual point values and median in relative fold. The horizontal line across individual values represents the median.

Furthermore, CD68 immunoreactivity of macrophages in coronary rupture plaques were noted (Figure 3(a); magnification, ×20), and strong visfatin immunoreactivity in atherosclerotic coronary rupture plaques from 7 patients with STEMI were found (Figure 3(c); magnification, ×20). Absence of staining was found in the control section when the primary antibody was replaced with nonimmune antiserum (Figures 3(b) and 3(d)). Furthermore, to further explore the potential role of visfatin in the pathogenesis of acute STEMI, circulating leukocytes with real-time PCR was conducted. Significant visfatin mRNA expression was observed in circulating leukocytes of acute STEMI patients than non-CAD controls (*P* = 0.003) (Figure 4).

## 4. Discussion

The predictive value of WBC counts in patients with AMI has been extensively reported in recent years [32–34] but rare research has been made regarding any specific subtype of leukocytes with adipocytokines that could be responsible for this detailed association. The present study indicates two major findings. First, we demonstrated that visfatin expressed in monocytes and neutrophils and increased their reactivity in male acute STEMI patients. Second, visfatin strongly appeared in lipid rich coronary rupture plaques and macrophages. These results suggest another explanation about how leukocytes mediated visfatin that may play a pathogenesis role in coronary vulnerable plaques rupture. 

One large study has reported that a high monocyte count in middle-aged healthy men was a predictor of coronary events during the followup [35]. Among the WBC fractions evaluated, only the monocyte count predicted the risk of coronary events. The inflammatory infiltrate process should be considered an independent expression of coronary disease severity in all plaques of AMI. Plaque inflammation has emerged as an obligatory feature in events leading to plaque vulnerability and rupture [36]. Plaque rupture coexists with numerous inflammatory cells and chemokines, mainly from macrophage foam cells [37]. Macrophages synthesize and release multiple growth factors, and also secrete metalloproteinases that weaken the fibrous cap and predispose it to rupture which may be the important mechanism of AMI [38]. Recent studies suggest that visfatin may be one of the clinically important cytokines associated with inflammation, atherosclerosis, and the role of plaque destabilization in ACS [23, 24]. It has been demonstrated that visfatin could activate human leukocytes expression of IL-1*β*, TNF-*α*, IL-6 [39] and increase monocyte matrix metalloproteinase-9 activity in monocytic THP-1 cells [24]. These mechanisms may partially explain our previous reports about plasma visfatin levels which are associated with IRA occlusion and closely related to the degree of myocardial damage in acute STEMI patients [25, 26]. However, the biological mechanisms involving intraleukocyte visfatin expression in the pathogenesis of STEMI are not well understood.

Previous study demonstrated that visfatin induces the expression of the costimulatory molecules CD80 (B7-1) and CD40 in human monocytes and observed a significant induction of intercellular adhesion molecule-1 (CD54) [39], another costimulatory ligand that binds to lymphocyte function- associated antigen-1, thereby promoting the activation of T-cells [40]. In addition, evidence showed that visfatin affects primary lymphocyte responses was demonstrated by an increased dose-dependent proliferative response after preincubating monocytes with visfatin [41]. In the present study, IHC stain qualified visfatin location and mRNA real-time PCR quantified visfatin expression reveals the partial mechanism of visfatin elevation in acute STEMI. We have demonstrated visfatin is dominantly localized in neutrophil and monocyte by IHC stain and enhanced expression by real-time PCR. Hence, in acute stage of AMI patients, serum visfatin levels elevation, one of the major sources is from neutrophil and monocyte. It can be explained that in the pre-PCI acute phase of the AMI group subjects, rapid neutrophil and monocyte counts production is correlated with visfatin levels elevated than those of non-AMI controls. 

In addition, our results also showed that visfatin was diffusely expressed in the coronary rupture plaques including in lipid core and many infiltrated macrophages besides the destruct endothelium and it was concurred to the previous study [24]. Furthermore, we also demonstrated that the visfatin mRNA expression in circulating leukocytes was significantly increased in acute STEMI patients than non-CAD control subjects. Maden et al. [42] and Barron et al. [43] found that elevated WBC count and activated leucocytes were associated with reducing epicardial blood flow and myocardial perfusion as well as thromboresistance, and playing a role in the pathophysiological process leading to IRA occlusion. On the basis of these observations, we think that inflammatory cells overexpression of visfatin in circulation and coronary rupture plaques may explain the growing body of literature that links inflammation and acute STEMI. We believe that further studies are required to ascertain the role of visfatin in patients presenting with AMI. 

The limitation of our study, whether elevated visfatin levels was found with STEMI can be translated directly from intracellular signal over-expression into endothelial dysfunction, vascular inflammation, plaque dysabilization, oxidative stress, coagulant activity increase, still need further efforts to be elucidated. 

In conclusion, male patients with STEMI showed increased visfatin expression in leukocytes, which may aggravate the development of instability of atherosclerotic plaques. Therefore, the leukocytes mediated visfatin expression may be a valuable marker for coronary vulnerable plaques rupture and may play a potentially pathological role in STEMI.
